# Associations of Cannabis and Cigarette Use with Depression and Anxiety at Age 18: Findings from the Avon Longitudinal Study of Parents and Children

**DOI:** 10.1371/journal.pone.0122896

**Published:** 2015-04-13

**Authors:** Suzanne H. Gage, Matthew Hickman, Jon Heron, Marcus R. Munafò, Glyn Lewis, John Macleod, Stanley Zammit

**Affiliations:** 1 School of Social and Community Medicine, University of Bristol, Bristol, United Kingdom; 2 Medical Research Council Integrative Epidemiology Unit at the University of Bristol, Bristol, United Kingdom; 3 United Kingdom Centre for Tobacco Control Studies, University of Bristol, Bristol, United Kingdom; 4 Mental Health Sciences Unit, University College London, London, United Kingdom; 5 Medical Research Council Centre for Neuropsychiatric Genetics and Genomics, Cardiff University, Cardiff, United Kingdom; Chiba University Center for Forensic Mental Health, JAPAN

## Abstract

**Introduction:**

Substance use is associated with common mental health disorders, but the causal effect of specific substances is uncertain. We investigate whether adolescent cannabis and cigarette use is associated with incident depression and anxiety, while attempting to account for confounding and reverse causation.

**Methods:**

We used data from ALSPAC, a UK birth cohort study, to investigate associations between cannabis or cigarettes (measured at age 16) and depression or anxiety (measured at age 18), before and after adjustment for pre-birth, childhood and adolescent confounders. Our imputed sample size was 4561 participants.

**Results:**

Both cannabis (unadjusted OR 1.50, 95% CI 1.26, 1.80) and cigarette use (OR 1.37, 95% CI 1.16, 1.61) increased the odds of developing depression. Adjustment for pre-birth and childhood confounders partly attenuated these relationships though strong evidence of association persisted for cannabis use. There was weak evidence of association for cannabis (fully adjusted OR 1.30, 95% CI 0.98, 1.72) and insufficient evidence for association for cigarette use (fully adjusted OR = 0.97, 95% CI 0.75, 1.24) after mutually adjusting for each other, or for alcohol or other substance use. Neither cannabis nor cigarette use were associated with anxiety after adjustment for pre-birth and childhood confounders.

**Conclusions:**

Whilst evidence of association between cannabis use and depression persisted after adjusting for pre-term and childhood confounders, our results highlight the difficulties in trying to estimate and interpret independent effects of cannabis and tobacco on psychopathology. Complementary methods are required to robustly examine effects of cannabis and tobacco on psychopathology.

## Background

A variety of mental health problems, including depression and anxiety, occur more commonly in substance users than non-users.[1–4] However, discerning causation from confounding, reverse causation, or selection or measurement bias in observational studies is problematic.[5] The cannabinoid system in the brain has been implicated in emotional regulation,[6] but biological mechanisms that might underlie the association between cannabis use and depression are not known, and indeed effects of cannabis may differ between the short and long term. For example, while people often report that cannabis acutely elevates their mood, long-term cannabis users show an increased risk of a number of adverse outcomes, including poorer education and social functioning,[7] which might be expected to increase risk of depression.

A recent systematic review and meta-analysis of longitudinal studies reported a 1.17-fold increase in odds of depression (95% CI 1.05, 1.30) in cannabis users, with a larger effect (OR = 1.62, 95% CI 1.21, 2.16) for heavy use,[8] and concluded that cannabis is associated with a modest increase in risk of developing depressive disorders. An earlier meta-analysis reported a similar association, but the authors argued that the majority of contributing studies did not adequately account for the possibility of reverse causation.[9] They also noted the wide variation in the number and type of confounders adjusted for. In particular, about half the studies included in their meta-analysis made no adjustment for alcohol or other drug use, which, given their potential depressogenic and anxiogenic effects during intoxication and withdrawal,[10, 11] strongly suggests the potential for residual confounding. Similar problems are likely to exist for determining the causal role of cannabis on anxiety.[9]

Tobacco use is also more common in people with depression and anxiety compared to the general population,[1, 3] and determining if these associations are causal is beset with the same difficulties as for cannabis. Whilst some studies indicate that smoking may be a self-medication behaviour,[12] other studies provide evidence in support of a biological causal mechanism for this association. For example, findings from animal studies suggest that nicotine effects on strengthening excitatory synapses on midbrain dopamine neurons might weaken coping mechanisms.[13]

The main aim of this study was to investigate the association between cannabis and cigarette use and incident depression and anxiety, whilst attempting to address issues relating to confounding, reverse causation and bias as robustly as we could. Furthermore, as cannabis and cigarette use are also associated with psychotic experiences (PEs), and we recently reported such associations within this sample,[14] we also examine the specificity of cannabis and cigarettes by comparing their effects on depression with those for PEs.

## Methods

### Participants

The Avon Longitudinal Study of Parents and Children (ALSPAC) is a prospective, population-based birth cohort study that recruited 14,541 pregnant women resident in Avon, UK, with expected delivery dates April 1 1991 to December 31 1992 (http://www.alspac.bris.ac.uk). Information has been collected on the participants and their offspring from over 60 questionnaires and 9 clinic assessments.[15] (http://www.bris.ac.uk/alspac/researchers/data-access/data-dictionary/). The current study is based on 4,561 individuals who completed the Revised Clinical Interview Schedule (CIS-R) at age 18. Ethical approval for the study was obtained from the ALSPAC Ethics and Law Committee and the Local Research Ethics Committee.

### Measures

#### Exposures

Cannabis and cigarette use at age 16 were measured via self-report questionnaire (N = 5,068 & N = 5,074 respectively). Cumulative cannabis use was coded as a 4 level category variable: ‘0 times’, ‘1–20 times’, ‘21–60 times’ and ‘more than 60 times’ (see S1 File for further details). Frequency of smoking was coded as a 4 level category variable: ‘non-smokers’, ‘experimenters’, ‘weekly smokers’ and ‘daily smokers’.

#### Outcomes

Depression and anxiety were assessed at age 18 using the CIS-R [16] via a self-administered computerised interview. The primary outcomes were binary variables of depression (mild, moderate or severe) and of anxiety disorder (any of generalised anxiety disorder, social phobia, specific phobia, panic disorder, or agoraphobia) derived from algorithms based on ICD-10 criteria for unipolar depression and anxiety disorders respectively (see S1 File for further details).

#### Confounders

Based on the literature for associations between both cannabis and cigarette use with depression and anxiety, we considered the following as potential confounders: a) pre-birth confounders (*family history of depression[17]* [binary measure assessed via maternal questionnaire], *maternal education[18]* [a 5-level categorical variable assessed via maternal questionnaire], *urban living* [urban/town/village/hamlet, obtained from postcode of residence], and *gender[19]*); and b) childhood confounders (*IQ* at age 8[20] [assessed via Wechsler Intelligence Scale for Children[21]], *borderline personality traits[22]* [an 8-level measure assessed via interview at age 11], *victimisation[23]* [a binary measure assessed at age 8 via interview], *peer problems[24]* [a 10-point scale assessed via the strengths and difficulties interview at age 8[25]], and *conduct disorder* trajectory group ages 4–13[26] [membership of one of 4 trajectory paths (early onset persistent/childhood-limited/adolescent onset/low) as described in[27]]). We also examined *alcohol use[28]* [using the Alcohol Use Disorders Identification Test (AUDIT) score ranging from 0 to 40, assessed via questionnaire[29]] and *other illicit drug use[30]* [a 3 level measure none/other drugs/stimulants assessed via questionnaire] at age 16 as potential confounders. Cigarette and cannabis use were also considered as confounders for each other.

### Statistical analysis

We carried out the analyses in STATA version 13 (Stata Corp LP, College Station, TX USA) and Mplus. We assessed exposure-outcome relationships before and after adjustment for confounders using logistic regression. We excluded individuals with depression (cut-off score of 11 on moods and feelings questionnaire (MFQ)[31, 32]) or anxiety (from DAWBA[33]) at age 16 from the relevant analyses, in order to minimise reverse causation. We examined the impact of confounding by comparing unadjusted estimates (model 1) with those adjusting for pre-birth confounders (model 2), and those further adjusted for childhood confounders (model 3). Further adjustment was made separately for age 16 cigarette use or cannabis use as appropriate (model 4a), alcohol use (model 4b), and other illicit drug use (model 4c). Finally, we also ran a fully adjusted analysis (model 5). This step-wise approach was used to elucidate the impact of different confounders on the associations.

We used a bivariate probit regression model in Mplus to investigate whether cannabis (or cigarettes) had similar (or different) effects on depression as they did on PEs, whilst taking into account their comorbidity. Using Wald tests, we compared a model where effect estimates for depression and PEs were allowed to differ with one where they were constrained to be the same. Probit estimates were converted to approximate odds ratios (ORs) to allow for easier interpretation. A binary measure of PEs (suspected or definite versus none) at age 18 was derived following a semi-structured interview conducted by trained psychologists.[34]

### Analytical sample

**Fig 1:**
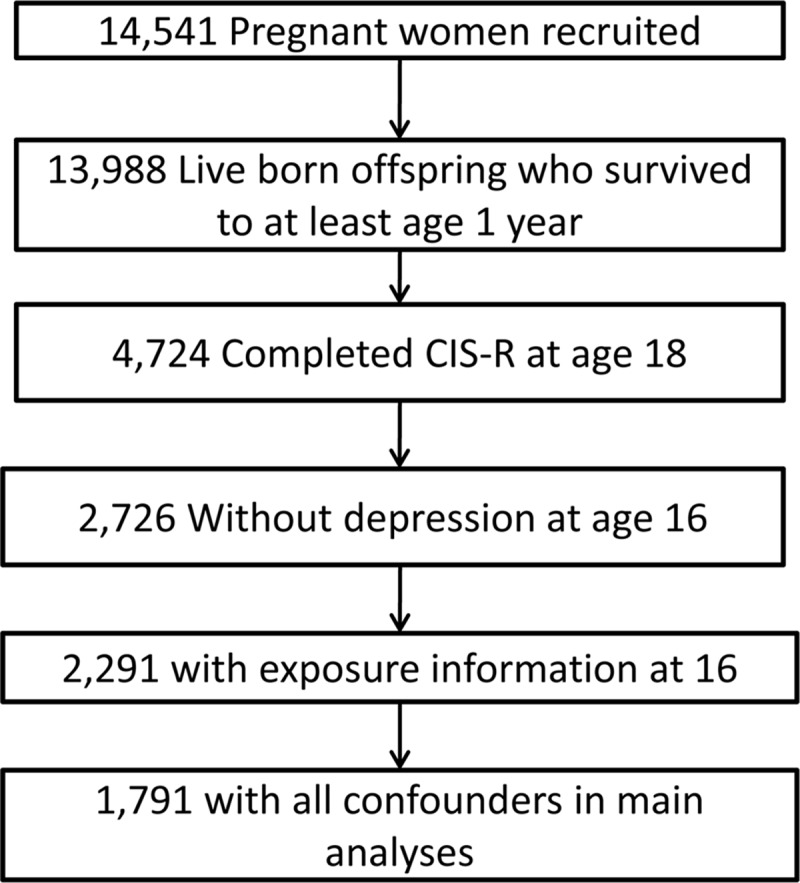
Study Participant Flow Diagram.

The complete sample with data on outcomes, exposures and confounders was 1791 (Fig 1). To address the potential bias introduced by attrition in our sample we conducted multiple imputation, creating 100 imputed datasets using additional information from over 50 variables associated with our observed measures and with missingness to make the assumption that data were ‘missing at random’ more plausible. The measures included related to pre-birth factors, childhood and adolescent behaviours, and earlier measures of psychopathology and substance use at various ages. Our primary results are presented using this sample with imputed confounder and exposure data (imputed sample N = 4561). There were 4345 people who had information on both depression and PEs at age 18, for the imputed bivariate analysis. Omitting those with previous depression or anxiety from imputed data is not trivial,[35] therefore the same estimates were obtained using a series of appropriately parameterized interaction models. Pre-existing depression or anxiety was included as a moderator variable in both the imputation and the analyses that followed. Given the number of interaction terms required, a separate imputation model was derived for each exposure/outcome pair in turn.

As a sensitivity analysis we assessed associations in the whole sample (N = 4561), without excluding those with depression at age 16. We also repeated the main analyses in the complete case dataset (N = 1512).

## Results

### Cannabis and depression

There were 128 people (7.2%, 95% CI 6.0, 8.4) who had depression at age 18, 94 females and 34 males. Of our sample with complete data, 491 (27.4%) had ever used cannabis by age 16, and 59 (3.3%) had used more than 60 times. Higher levels of cannabis use were more common in participants whose mothers had not undertaken higher education, in those with conduct disorder and who used other substances, and in those with a higher childhood IQ (Table 1).

**Table 1:** Descriptives of confounders by cumulative cannabis use at age 16% (N).

Cumulative cannabis age 16	0 times 72.4 (1300)	<20 times 20.3 (367)	21–60 times 3.8 (65)	>60 times 3.6 (59)	P value
FH depression	26.2 (341)	29.4 (108)	36.9 (24)	32.2 (19)	0.039
FH schizophrenia	0.8 (10)	1.1 (4)	0	1.7 (1)	0.622
Low Maternal education^1^	77.7 (1010)	70.3 (258)	61.5 (40)	71.2 (42)	0.001
Urban dwelling	89.2 (1159)	87.5 (321)	89.2 (58)	88.1 (52)	0.573
Gender (female)	57.4 (746)	65.1 (239)	53.9 (35)	45.8 (27)	0.787
Borderline personality^2^	1.3 (17)	1.1 (4)	1	1.7 (1)	0.700
IQ at age 8 m(sd)	109.1 (15.2)	111.9 (15.6)	115.7 (14.9)	111.9 (13.9)	<0.001
Psychotic experiences age 12	3.4 (44)	4.6 (17)	0	5.1 (3)	0.732
Conduct disorder^3^	5.2 (68)	9.3 (34)	7.7 (5)	15.3 (9)	<0.001
Peer problems (4 or more)	7.1 (93)	5.2 (19)	10.8 (7)	8.5 (5)	0.225
Bullied	30.3 (394)	29.7 (109)	29.2 (19)	32.2 (19)	0.976
Cigarette smoking age 16^4^	2.3 (30)	21.3 (78)	40.0 (26)	71.2 (42)	<0.001
Illicit drugs age 16	6.2 (80)	32.7 (120)	63.1 (41)	81.4 (48)	<0.001
Alcohol use age 16^5^	20.9 (272)	62.9 (231)	61.5 (40)	84.8 (50)	<0.001

FH- family history; IQ- intelligence quotient.

^1^No higher education. Categorised like this for purposes of presentation in this table only.

^2^Defined as score of 4 or over, for the purposes of presentation in this table only.

^3^Early onset persistent group membership. Categorised like this for purposes of presentation in this table only.

^4^Weekly or daily smoking. Categorised like this for purposes of presentation in this table only.

^5^Hazardous alcohol use. Categorised like this for purposes of presentation in this table only.

In the unadjusted analysis there was a 1.5-fold increase in the odds of depression per category increase of cumulative cannabis use (95% CI 1.26, 1.80). Adjustment for childhood confounders attenuated the association by approximately 20% though strong evidence of an association remained (Table 2). Further adjustment for adolescent substance use led to further attenuation in the size of the association (fully adjusted OR 1.30, 95% CI 0.98, 1.72). This implies an increased risk of depression of 2.2 in the highest category of self-reported cannabis use (> 60 times) versus never users.

**Table 2:** Logistic regression of intensity of cannabis or cigarette use at age 16 and Depression at age 18 using imputed data (100 imputations), accounting for those with depression at age 16* (N = 4561).

Model	Cannabis			Cigarettes		
	OR	95% CI	P value	OR	95% CI	P value
1	1.50	1.26, 1.80	<0.001	1.37	1.16, 1.61	<0.001
2	1.52	1.26, 1.82	<0.001	1.29	1.09, 1.53	0.003
3	1.41	1.17, 1.71	<0.001	1.20	1.01, 1.43	0.033
4a	1.32	1.03, 1.68	0.027	1.11	0.90, 1.38	0.331
4b	1.35	1.09, 1.67	0.006	1.15	0.95, 1.40	0.155
4c	1.38	1.09, 1.75	0.007	1.15	0.95, 1.40	0.153
5	1.30	0.98, 1.72	0.065	1.09	0.87, 1.36	0.460

Model 1—Case depression at 18 by unit increase of 4-level categorical cumulative cannabis or frequency of cigarette use at 16.

Model 2—as model 1 with additional adjustment for pre birth confounders (family history of depression, gender, urban dwelling, maternal education).

Model 3—as model 2 with additional adjustment for childhood confounders (borderline personality, IQ at age 8, PEs at age 12, conduct disorder trajectory group membership, peer problems, bullied).

Model 4a —as model 3 with additional adjustment for cigarette use/cannabis use (as appropriate).

Model 4b —as model 3 with additional adjustment for alcohol use.

Model 4c —as model 3 with additional adjustment for illicit drug use (other than cannabis).

Model 5—as model 3 with additional adjustment for cigarette (or cannabis), alcohol, other illicit drug use.

* The imputed interaction model allowed us to account for 15% of participants who reported depression at age 16, without exclusions.

### Cigarette use and depression

Of those with complete data, 803 (44.8%) had ever smoked a cigarette, with 99 (5.5%) smoking every day. Higher frequency of cigarette use occurred in females, and in those with conduct disorder and those who used other substances (Table 3).

**Table 3:** Descriptives of confounders by frequency of cigarette use at age 16% (N).

Cigarette frequency age 16	Never 55.2 (988)	Experimenter 35.0 (627)	Weekly 4.3 (77)	Daily 5.5 (99)	P value
FH depression	25.8 (255)	28.9 (181)	28.6 (22)	34.4 (34)	0.044
FH schizophrenia	0.9 (9)	0.8 (5)	0	1.0 (1)	0.742
Maternal education^1^	74.8 (739)	75.0 (470)	79.2 (61)	80.8 (80)	0.189
Urban dwelling	88.7 (876)	89.0 (558)	90.9 (70)	86.9 (86)	0.925
Gender (female)	51.0 (504)	67.5 (423)	72.7 (56)	64.7 (64)	<0.001
Borderline personality^2^	1.0 (10)	1.6 (10)	0	2.0 (2)	0.467
IQ at age 8 m(sd)	109.9 (15.2)	110.8 (15.3)	108.6 (14.2)	106.6 (16.7)	0.199
Psychotic experiences age 12	3.0 (30)	4.2 (26)	2.6 (2)	6.1 (6)	0.138
Conduct disorder^3^	5.3 (52)	7.0 (44)	9.1 (7)	13.1 (13)	0.001
Peer problems	7.6 (75)	5.7 (36)	1.3 (1)	12.1 (12)	0.661
Bullied	30.5 (301)	30.0 (188)	27.3 (21)	31.3 (31)	0.868
Cannabis age 16^4^	5.0 (49)	47.2 (296)	73.3 (58)	88.9 (88)	<0.001
Illicit drugs age 16	5.2 (51)	22.5 (141)	44.2 (34)	63.6 (63)	<0.001
Alcohol use age 16^5^	15.3 (151)	50.7 (318)	67.5 (52)	72.7 (73)	<0.001

^1^No higher education. Categorised like this for purposes of presentation in this table only.

^2^Defined as score of 4 or over, for the purposes of presentation in this table only.

^3^Early onset persistent group membership. Categorised like this for purposes of presentation in this table only.

^4^Ever used cannabis. Categorised like this for purposes of presentation in this table only.

^5^Hazardous alcohol use. Categorised like this for purposes of presentation in this table only.

Cigarette use at age 16 was associated with an increased odds of depression at age 18 (unadjusted OR per category of cigarette use = 1.37, 95% CI 1.16, 1.61). Adjusting for pre-birth and childhood confounders attenuated the association by about 50%, though some evidence of an association persisted (Table 2). Further adjusting for cannabis use eliminated evidence of association between cigarette use and depression, with adjustment for alcohol, or illicit drug use, resulting in similar (though less strongly attenuated) results (fully adjusted OR = 0.97, 95% CI 0.75, 1.24). Cigarette use and cannabis use were correlated (polychoric rho = 0.78, SE 0.01).

### Bivariate Analysis

Adjustment for childhood confounders and substance use also attenuated the association between cannabis or tobacco and psychotic experiences. There was no evidence (Table 4) of differences between i) the association between cannabis and depression, and that between cannabis and PEs (Model 5 Wald test -0.010, p = 0.913), or ii) the association between cigarette use and depression, and that between cigarettes and PEs (Model 5 Wald test -0.043, p = 0.523).

**Table 4:** Bivariate probit regression analysis of intensity of cannabis or cigarette use at 16 and depression and PEs at age 18 in the imputed dataset.

N = 4345	Depression			Psychotic Experiences			
	OR	95% CI	p	OR	95% CI	p	Wald test (p value)
	Cannabis						
1	1.34	1.18, 1.51	<0.001	1.38	1.23, 1.55	0.001	-0.023 (0.637)
3	1.29	1.13, 1.47	<0.001	1.41	1.24, 1.59	<0.001	-0.056 (0.292)
5	1.11	0.89, 1.38	0.343	1.12	0.91, 1.39	0.260	-0.010 (0.913)
	Cigarettes						
1	1.37	1.23, 1.53	<0.001	1.42	1.28, 1.57	<0.001	-0.018 (0.654)
3	1.24	1.10, 1.39	<0.001	1.33	1.19, 1.49	<0.001	-0.046 (0.307)
5	1.08	0.91, 1.28	0.367	1.15	0.98, 1.36	0.069	-0.043 (0.523)

Model 1—Case depression at 18 and suspected/definite PEs at 18 by unit increase of 4-level categorical cumulative cannabis use or frequency of cigarette use at 16.

Model 3—as model 2 with additional adjustment for childhood confounders (borderline personality, IQ at age 8, PEs at age 12, conduct disorder trajectory group membership, peer problems, bullied).

Model 5—as model 3 with additional adjustment for cigarette (or cannabis), alcohol and other illicit drug use.

### Anxiety

There were 162 (9.0%, 95% CI 7.8, 10.5) participants in our sample with anxiety at age 18. There was weak evidence of an association between cannabis use and anxiety disorder at age 18, but no evidence after adjustment for pre-birth and childhood confounders (S1 Table), and further attenuation still after adjusting for substance use (unadjusted OR = 1.13, 95% CI 0.98, 1.31; fully adjusted OR = 0.96, 95% CI 0.75, 1.24). The same pattern of association was seen between cigarette use and anxiety (unadjusted OR = 1.14, 95% CI 1.00, 1.30; fully adjusted OR = 0.85, 95% CI 0.69, 1.04).

### Sensitivity analyses

In the complete case analysis, associations between cannabis and depression were of a larger magnitude than in the imputed sample (S2 Table). Adjustment for cigarette use increased the estimate size, and the fully adjusted OR was larger than the unadjusted. Associations between cigarette use and depression were broadly similar to the imputed sample. Associations between cannabis and anxiety and between cigarettes and anxiety were also broadly similar to the imputed sample (S3 Table).

## Discussion

Intensity of cannabis use at age 16 was associated with an increased incidence of depression at age 18. Adjusting for confounders assessed pre-birth and during childhood partly attenuated the association with depression, although strong evidence for an association with depression persisted. Further adjustment for cigarette, alcohol, or other drug use weakened the evidence of association with depression, but also widened the confidence intervals around the effect estimates. The strong correlation between cannabis use and other substances, particularly cigarette use, may have led to collinearity problems with the statistical model, as evinced by the increase in the size of the standard errors, and makes it difficult to interpret the results of cannabis independently from those for other substance use.

In addition, investigation of cannabis users who claimed not to smoke cigarettes in our study revealed that the vast majority of them (44 out of 47) used tobacco with their cannabis and were thus still exposed to tobacco.[14] Therefore, our study (and almost certainly other studies in countries where cannabis is smoked with tobacco that have adjusted for cigarette smoking) will not have adequately adjusted for tobacco use. A further difficulty in interpreting results adjusted for cigarette or other substance use is that it is feasible that use of these substances occurred secondary to the use of cannabis, for example through exposure to situations where tobacco, alcohol or other drugs became more readily available. This is an example of “collider bias”[36] and could have biased estimates of the association between cannabis and depression.[37]

Cigarette use was also associated with an increased incidence of depression. However, adjusting for confounders measured pre-birth and during childhood attenuated the association with depression to a greater extent than they did for cannabis and depression. Further adjustment for cannabis or other substance use eliminated this association, though the same problem of potential collider bias exists here too.

In contrast to our findings for depression, there was only weak evidence of associations between cannabis or cigarettes and anxiety, and these associations were eliminated after adjusting for pre-birth and childhood confounders.

Whilst most results were similar in the sensitivity analysis using our smaller, non-imputed (complete case) sample, one exception was that the association between cannabis and depression *increased* after adjustment for cigarette use. One explanation for this difference is that our complete case analysis is biased as a result of non-differential attrition. Indeed the direction of change in the estimate after adjusting for cigarette use is opposite to that expected given the relationship between cigarette use and depression, and that between cigarette use and cannabis. Our multiple imputation analysis is likely to be correcting for this to some extent as our imputation model was carefully designed to try and accurately predict the missingness in our variables.

The effect sizes we observed are consistent with previous meta-analyses that have reported associations between cannabis use and later depression of a moderate-sized effect.[8, 9] Furthermore, whilst we do not find evidence to support an association between cannabis use and incident anxiety we cannot exclude a small effect compatible with confidence limits from a recent meta-analysis of association between cannabis use and anxiety.[38] Although authors of that study attempted to assess the quality of included studies, estimating causal effect sizes from meta-analyses of observational studies are inherently limited by inclusion of studies with sub-optimal adjustment for confounding. For example, in Moore and colleagues’ meta-analysis attenuation of unadjusted estimates was greater in studies that adjusted for the most comprehensive sets of confounders.[9]

Whilst research from post-mortem human brain,[39] neuroimaging,[40] and animal[41] studies provide some evidence of plausible mechanisms by which cannabis might cause depression, this body of evidence is by no means compelling. For example, evidence from animal models suggests that tetrahydrocannabinol (THC: a cannabinoid receptor type 1 (CB1) agonist) during adolescence increases adult anhedonia and other models of depression and anxiety,[42, 43] but there is also evidence that rimonabant (a CB1 antagonist) increases anxiety, depression and suicidality.[44] Differences between acute and longer term effects of cannabis on the brain have yet to be clearly elucidated, and have to incorporate evidence of euphoria during acute intoxication with long-term adverse outcomes in cannabis users such as impaired occupational and social function that might be expected to increase depression (though studies of these outcomes are subject to the same problems of residual confounding as studies of depression). Furthermore, whilst studies comparing the effects of cannabis strains with different concentrations of THC, or different ratios of THC to cannabidiol (a cannabinoid that acts as a CB1 antagonist) on depression and anxiety can potentially help with understanding of the causal role of cannabis on these disorders, there are few such studies to date.[45]

Despite a common impression that the evidence supporting a causal effect of cannabis on psychosis is stronger than that for depression we found no evidence that associations between cannabis or cigarette use and depression were different to those between cannabis or cigarette use and PEs in our bivariate analysis. Whilst it is possible that we did not have adequate power to detect a difference, nevertheless our results suggest that associations between substance use and mental health, if causal, seem to be non-specific, both in terms of substances and outcomes.

There are a number of important limitations to our study. First, wave non-response and attrition (almost inevitable in a longitudinal study of this nature) means that our sample was small compared to the original ALSPAC sample, and power to detect small effects was limited; nevertheless our sample was still larger than most other cohorts that have examined the research questions we address here,[9] and we used multiple imputation to reduce attrition bias. It is possible that our imputation did not adequately address the missing at random assumption and that bias from selective attrition persisted, or that higher-order confounding was present and inadequately represented in the imputed models. However, imputed models are still likely to be less biased than use of complete case data. Our exposure measures were based on self-report data, which might be a limitation particularly for illicit drug use. If, for example, proneness to depression made individuals more reluctant to admit to illegal drug use this may have led to us underestimating the association between cannabis use and depression, though we have no reasons to suspect this occurred. Our outcome measures were assessed via computerised interview, therefore may not fully have captured depression or anxiety in the participants. However, prevalence was equivalent to that expected from previous literature [46, 47]. Finally, the problem of co-occurrence of cannabis and tobacco in this and other studies to date has already been discussed above, and would not be improved substantially by increasing sample size or having more accurate measures of substance use. Identifying populations where individuals use cannabis without mixing it with tobacco may help address this limitation, though we are not aware of any longitudinal studies in such populations currently. Utilising genetic variants associated with tobacco or cannabis use as instrumental variables in Mendelian randomisation analyses offers a potential approach for determining whether cannabis or tobacco have causal effects on psychopathology, but is limited by requirement of samples larger than currently available,[48] and by the absence of genetic proxies for cannabis use.[49]

## Conclusions

Whilst most associations we examined were explained to a substantial degree by confounders assessed pre-term or during childhood, strong evidence of association between cannabis use and depression persisted. However, interpreting results for cannabis or cigarette use independent of each other is problematic, and our study highlights the limitations inherent in observational studies that aim to determine whether cannabis or tobacco use have causal effects on psychopathology. Novel approaches are required to determine whether associations between cannabis or tobacco and psychopathology are causal, and to help inform public knowledge and health prevention policy.

## Supporting Information

S1 File Supporting information showing the questions asked regarding cannabis, cigarette use and depression. (DOCX)

S1 Table Logistic regression of intensity of cannabis or cigarette use at age 16 and Anxiety at age 18 in imputed datasets, accounting for those with anxiety at age 15* (N = 4561). (DOCX)

S2 Table Logistic regression of intensity of cannabis or cigarette use at age 16 and Depression at age 18 in CCA, excluding those with depression at age 16 (N = 1512). (DOCX)

S3 Table Logistic regression of intensity of cannabis or cigarette use at age 16 and Anxiety at age 18 in CCA, excluding those with anxiety at age 15 (N = 1682). (DOCX)
