# Supplementary material for: Associations of Cannabis and Cigarette Use with Depression and Anxiety at Age 18: Findings from the Avon Longitudinal Study of Parents and Children
Source: PLoS One. 2015 Apr 13;10(4):e0122896. doi: 10.1371/journal.pone.0122896 (PMC4395304; doi:10.1371/journal.pone.0122896)
Supplement: S1 File — (DOCX) [file pone.0122896.s001.docx]

Appendix

Questions asked:

Cannabis use:

How many times have you taken cannabis in total? 6 options: ‘never’, ‘less than 5 times’, ‘5-20 times’, ’21-60 times’, ’61-100 times’ and ‘more than 100 times’

Tobacco use:

Please mark the box that describes you best? 7 options: ‘I have never smoked a cigarette’, ‘I have only smoked a cigarette once or twice’, ‘I used to smoke sometimes but I never smoke cigarettes now’, ‘I sometimes smoke cigarettes but I smoke less than once a week’, ‘I usually smoke between 1 and 6 cigarettes a week’, ‘I usually smoke more than 6 cigarettes a week, but not every day’ or ‘I usually smoke one or more cigarettes every day’.

Depression:

To meet criteria the subject must have reported at least one of two ‘entry criteria’ symptoms for at least 2 weeks. The entry criteria symptoms are: experiencing loss of pleasure and experiencing persistent low mood. At least 2 of 7 possible associated symptoms are also required for a diagnosis (disturbed appetite, sleep problems, impaired motor responses, poor concentration, fatigue, poor self-esteem/negative cognitions and suicidality).
