# Supplementary material for: Associations of Cannabis and Cigarette Use with Depression and Anxiety at Age 18: Findings from the Avon Longitudinal Study of Parents and Children
Source: PLoS One. 2015 Apr 13;10(4):e0122896. doi: 10.1371/journal.pone.0122896 (PMC4395304; doi:10.1371/journal.pone.0122896)
Supplement: S1 Table — (DOCX) [file pone.0122896.s002.docx]

Table S1: Logistic regression of intensity of cannabis or cigarette use at age 16 and Anxiety at age 18 in imputed datasets, accounting for those with anxiety at age 15* (N=4561)

|  | Cannabis | | | Cigarettes | |  |
| --- | --- | --- | --- | --- | --- | --- |
| Model | OR | 95% CI | P value | OR | 95% CI | P value |
| 1 | 1.13 | 0.98, 1.31 | 0.090 | 1.14 | 1.00, 1.30 | 0.058 |
| 2 | 1.13 | 0.98, 1.31 | 0.090 | 1.06 | 0.92, 1.21 | 0.441 |
| 3 | 1.02 | 0.87, 1.19 | 0.852 | 0.95 | 0.82, 1.09 | 0.458 |
| 4a | 1.08 | 0.88, 1.34 | 0.460 | 0.90 | 0.74, 1.08 | 0.256 |
| 4b | 0.97 | 0.80, 1.17 | 0.727 | 0.89 | 0.75, 1.05 | 0. 159 |
| 4c | 0.92 | 0.75, 1.13 | 0.422 | 0.88 | 0.74, 1.04 | 0.122 |
| 5 | 0.96 | 0.75, 1.24 | 0.773 | 0.85 | 0.69, 1.04 | 0.107 |

Model 1 – Case depression at 18 by unit increase of 4-level categorical cumulative cannabis use or frequency of cigarette use at 16

Model 2 – as model 1 with additional adjustment for pre birth confounders (family history of depression, gender, urban dwelling, maternal education)

Model 3 – as model 2 with additional adjustment for childhood confounders (borderline personality, IQ at age 8, PEs at age 12, depression at age 12, conduct disorder trajectory group membership, peer problems, bullied)

Model 4a – as model 3 with additional adjustment for cigarette use (or cannabis use, as appropriate)

Model 4b – as model 3 with additional adjustment for alcohol use

Model 4c – as model 3 with additional adjustment for illicit drug use (other than cannabis)

Model 5 – as model 3 with additional adjustment for cigarette (or cannabis), alcohol and other illicit drug use

* The interaction model allowed us to account for 2.3% of participants deemed to have anxiety at age 15 without exclusions
