# Supplementary material for: Associations of Cannabis and Cigarette Use with Depression and Anxiety at Age 18: Findings from the Avon Longitudinal Study of Parents and Children
Source: PLoS One. 2015 Apr 13;10(4):e0122896. doi: 10.1371/journal.pone.0122896 (PMC4395304; doi:10.1371/journal.pone.0122896)
Supplement: S2 Table — (DOCX) [file pone.0122896.s003.docx]

Table S2: Logistic regression of intensity of cannabis or cigarette use at age 16 and Depression at age 18 in CCA, excluding those with depression at age 16 (N=1512)

|  | Cannabis | | | Cigarettes |  |  |
| --- | --- | --- | --- | --- | --- | --- |
| Model | Odds Ratio | 95% CI | P value | Odds Ratio | 95% CI | P value |
| 1 | 1.62 | 1.25, 2.09 | <0.001 | 1.34 | 1.04, 1.72 | 0.022 |
| 2 | 1.66 | 1.28, 2.15 | <0.001 | 1.28 | 0.99, 1.65 | 0.061 |
| 3 | 1.64 | 1.26, 2.15 | <0.001 | 1.21 | 0.93, 1.57 | 0.158 |
| 4a | 1.91 | 1.32, 2.77 | 0.001 | 0.85 | 0.59, 1.21 | 0.359 |
| 4b | 1.56 | 1.15, 2.12 | 0.004 | 1.07 | 0.79, 1.45 | 0.651 |
| 4c | 1.78 | 1.25, 2.54 | 0.001 | 1.11 | 0.82, 1.49 | 0.514 |
| 5 | 2.00 | 1.29, 3.09 | 0.002 | 0.82 | 0.56, 1.18 | 0.282 |

Model 1 – Case depression at 18 by unit increase of 4-level categorical cumulative cannabis use/frequency of cigarette use at 16

Model 2 – as model 1 with additional adjustment for pre birth confounders (family history of depression, gender, urban dwelling, maternal education)

Model 3 – as model 2 with additional adjustment for childhood confounders (borderline personality, IQ at age 8, PEs at age 12, conduct disorder trajectory group membership, peer problems, bullied)

Model 4a – as model 3 with additional adjustment for cigarette use (or cannabis, as appropriate)

Model 4b – as model 3 with additional adjustment for alcohol use

Model 4c – as model 3 with additional adjustment for illicit drug use (other than cannabis)

Model 5 – as model 3 with additional adjustment for cigarette (or cannabis), alcohol, other illicit drug use and depression score at age 16
