# Supplementary material for: Associations of Cannabis and Cigarette Use with Depression and Anxiety at Age 18: Findings from the Avon Longitudinal Study of Parents and Children
Source: PLoS One. 2015 Apr 13;10(4):e0122896. doi: 10.1371/journal.pone.0122896 (PMC4395304; doi:10.1371/journal.pone.0122896)
Supplement: S3 Table — (DOCX) [file pone.0122896.s004.docx]

Table S3: Logistic regression of intensity of cannabis or cigarette use at age 16 and Anxiety at age 18 in CCA, excluding those with anxiety at age 15 (N=1682)

|  | Cannabis | | | Cigarettes | |  |
| --- | --- | --- | --- | --- | --- | --- |
| Model | OR | 95% CI | P value | OR | 95% CI | P value |
| 1 | 1.17 | 0.93, 1.47 | 0.191 | 1.06 | 0.86, 1.31 | 0.593 |
| 2 | 1.17 | 0.92, 1.48 | 0.193 | 1.00 | 0.81, 1.25 | 0.966 |
| 3 | 1.08 | 0.84, 1.38 | 0.541 | 0.96 | 0.77, 1.20 | 0.715 |
| 4a | 1.20 | 0.87, 1.65 | 0.259 | 0.87 | 0.66, 1.17 | 0.360 |
| 4b | 1.05 | 0.79, 1.38 | 0.755 | 0.90 | 0.69, 1.17 | 0.419 |
| 4c | 0.94 | 0.69, 1.29 | 0.724 | 0.85 | 0.66, 1.10 | 0.208 |
| 5 | 1.06 | 0.73, 1.53 | 0.773 | 0.82 | 0.60, 1.10 | 0.189 |

Model 1 – Case depression at 18 by unit increase of 4-level categorical cumulative cannabis use or frequency of cigarette use at 16

Model 2 – as model 1 with additional adjustment for pre birth confounders (family history of depression, gender, urban dwelling, maternal education)

Model 3 – as model 2 with additional adjustment for childhood confounders (borderline personality, IQ at age 8, PEs at age 12, depression at age 12, conduct disorder trajectory group membership, peer problems, bullied)

Model 4a – as model 3 with additional adjustment for cigarette use (or cannabis use, as appropriate)

Model 4b – as model 3 with additional adjustment for alcohol use

Model 4c – as model 3 with additional adjustment for illicit drug use (other than cannabis)

Model 5 – as model 3 with additional adjustment for cigarette (or cannabis), alcohol and other illicit drug use
